# Chromatin Profiling Reveals Distinct Male and Female Trajectories for Developmental Learning Potential

**DOI:** 10.1002/dneu.23017

**Published:** 2025-11-10

**Authors:** Grant W. Kunzelman, Alice Batistuzzo, Sarah E. London

**Affiliations:** ^1^ Integrative Neuroscience Graduate Program Chicago Illinois USA; ^2^ Department of Psychology University of Chicago Chicago Illinois USA; ^3^ Institute for Mind and Biology Chicago Illinois USA; ^4^ Neuroscience Institute Chicago Illinois USA

**Keywords:** critical period, epigenetics, H3K27ac, learning and memory, regulatory regions, sensitive period, songbird

## Abstract

Adult patterns of behavior can often be explained by developmental experiences. In some cases, developmental experience can have permanent influence on brain and behavior only during specific ages; these phases are called critical or sensitive periods. Epigenetic mechanisms can regulate both maturational and experiential processes in the brain by coordinating transcription of genes involved in organization and plasticity. Epigenetics thus may have particular relevance to critical periods. As such, we employed ChIP‐seq to assess accessible regulatory regions, segments of the genome where transcription factors (TFs) bind, using the epigenetic marker H3K27ac. We focused on the auditory forebrain, required for developmental sensory song learning, in juvenile male and female zebra finches (*Taeniopygia guttata*). Both sexes rely on developmental sensory learning to bias adult behaviors, though males have a defined critical period for this process, whereas it is not clear that females do. Thus, we sought to address two major questions: (1) Are H327ac peaks changing in males as they transition into their critical period, and if so, how?, and (2) How similar are the female H3K27ac peaks at the same ages of development? Our analyses revealed that age and sex affect H3K27ac‐based peak profiles and enriched TF binding sites within them, as well as genes annotated to those H3K27ac‐defined peaks. These findings provide new insights into how epigenetic regulation may influence auditory forebrain organization and function in the context of changing learning potential across a sensitive developmental period and create a foundation for additional studies.

## 1 Introduction

Developmental learning can have life‐long consequences for neural function and behavior. However, the ability to learn is not consistent across development—there can be phases when specific experience can have maximal and permanent influence over brain function and behavior. These phases, called critical or sensitive periods, indicate the existence of shifting neural states that are more or less effective at encoding experience. Despite extensive knowledge of adult learning and memory mechanisms, we have scant information about what limits and promotes learning ability across development, which requires a consideration of age. A deeper understanding of these developmental dynamics may uncover age‐specific susceptibilities in neural circuits, enabling targeted investigations to enhance or minimize the effects of experience during development.

Here, we use a model that has an established critical period for learning to expand our understanding of neural properties that might determine the potential to learn from experience. Male zebra finches (*Taeniopygia guttata*) use the memory of an adult “tutor” song they acquire between the ages of posthatch day (P) 30 and 65 as a template for their own adult song structure (Eales 1985, 1987; Slater et al. 1991; Morrison and Nottebohm 1993; Slater et al. 1993; Roper and Zann 2006). Because adult males sing all day every day, the restricted period reflects a change in the potential to learn, not in access to relevant experience. Female zebra finches cannot sing, but they also perform juvenile sensory song learning that influences their adult song preferences (Miller 1979a; Clayton 1988, 1990; Riebel 2000, 2003a, 20032003b, 2009; Riebel et al. 2002; Lauay et al. 2004; Terpstra et al. 2006; Chen et al. 2017; Diez et al. 2019). The ages during which females can learn overlap with the defined sensitive period in males, though it is not clear if these ages are as restricted as in males (Miller 1979a, 1979b; Clayton, 1988, 1990; Riebel 2000, 2003a; Riebel et al. 2002; Braaten 2010; Holveck and Riebel 2014). Molecular and functional tests indicate that juvenile sensory song learning requires the auditory forebrain in both sexes (Jin and Clayton 1997; Bolhuis et al. 2000; Mello et al. 2004; Lauay et al. 2005; Terpstra et al. 2006; Tomaszycki et al. 2006; London and Clayton 2008; London et al. 2009; Yanagihara and Yazaki‐Sugiyama 2016; Ahmadiantehrani and London 2017; Lampen et al. 2017; Diez et al. 2019). However, despite no obvious morphological sex differences in the auditory forebrain, there is evidence that even at the same age, juvenile males and females may learn using distinct mechanisms during the male sensitive period (Clayton 1988; Riebel 2000; Bailey and Wade 2003; Riebel 2009; Derégnaucourt et al. 2013; Holveck and Riebel 2014; Ahmadiantehrani and London 2017).

In the present study, we sought to characterize baseline molecular features of the auditory forebrain to identify organizational properties that may influence the capacity for learning. Rather than focusing on individual genes or proteins as in previous work, we employed chromatin immunoprecipitation followed by DNAseq (ChIP‐seq), a discovery tool that enables genome‐wide investigation. We performed H3K27ac (acetylated histone H3 lysine 27) ChIP‐seq to identify accessible regulatory regions, including putative proximal promoters and enhancers (Heintzman et al. 2007; Wang et al. 2008; Creyghton et al. 2010). Regulatory regions inform on transcription factors (TFs) that can coordinate gene expression; TFs act as “hub” genes, those that are positioned to influence sets of genes that collectively can modulate neural structure and function. We assayed male and female auditory forebrain at two ages, guided by the onset of the male sensitive period at P30, and when we have seen sex differences in molecular learning responses after song experience (Roper and Zann 2006; Ahmadiantehrani and London 2017). We performed a series of analyses to identify accessible regulatory regions that differ based on age and sex, predict TF binding sites (TFBS) present and enriched within these regions, and identify the genes that may be affected. Our results provide new specificity for the players that may be coordinating the transition into the critical period for males and thus may facilitate the onset of learning potential. Additionally, we contribute to the evidence that the juvenile auditory forebrain has distinct mechanisms that support learning in males and females. These data thus provide a foundation for future mechanistic investigations into developmental learning.

## 2 Methods

### 2.1 Animals and Housing

All procedures were conducted in accordance with the NIH guidelines for the care and use of animals for experimentation and were approved by the University of Chicago Institutional Animal Care and Use Committee (ACUP no. 72220). All experimental birds were hatched in the London laboratory breeding colony at the University of Chicago. Animals were housed on a 14:10 h light:dark cycle. Food and water were provided ad libitum. To test the effect of age and sex on chromatin profiles, males and females were reared in “Normal” conditions; they spent the entirety of their lives in the communal aviaries in which they hatched. Male and female birds were collected at two ages: post‐hatch (P) Day 23 (P23) and P30 (Figure S1).

### 2.2 Auditory Forebrain Collections

Auditory forebrain collections for ChIP‐seq were performed between 6‐ and 8‐h post‐lights‐on. Auditory forebrains were dissected within 2 min of sacrifice, and the bilateral tissues were immediately flash‐frozen using dry ice. Samples were collected between August 10, 2019 and September 29, 2019. All tissue was stored at −80°C until ChIP processing, beginning October 13, 2019. To obtain enough chromatin to perform successful ChIP‐seq for H3K27ac, each analyzed sample was a pool of auditory forebrains from three individuals. There were *n* = 3 pooled samples for all four ChIP‐seq groups: P23 males (P23M), P23 females (P23F), P30 males (P30M), and P30 females (P30F). For RNA Sequencing (RNAseq), an independent set of P23M, P23F, P30M, and P30F auditory forebrain samples were collected as part of a larger experiment. To obtain baseline RNA measures, birds were placed in an acoustic isolation chamber overnight at either P22 or P29. After 6–8 h, the lights turned on the next morning, and auditory forebrains were dissected and rapidly frozen on dry ice and stored at −80°C until processing. No pooling was performed; each RNAseq sample corresponded to a single individual (*n* = 4 for all groups). The sex of all birds was confirmed by visual inspection of the testes or ovary. To avoid introducing bias based on genetic relatedness or parental behavior, no biological pool (ChIP‐seq) or group (RNAseq) contained tissue from siblings.

### 2.3 Chromatin Immunoprecipitation and Sequencing (ChIP‐seq)

ChIP‐seq for H3K27ac histone modifications was performed by the Service Department at Active Motif using a previously validated antibody for immunoprecipitation (Active Motif Cat# 39133; Carlsbad, CA). A pool of DNA consisting of equal proportions from all samples, but not immunoprecipitated, served as input control. A total of 75 nt single‐end reads were acquired with Illumina NextSeq 500.

### 2.4 ChIP‐Seq Read Quality Assessment and Genomic Alignment

DNAseq reads were quality‐checked and trimmed using FastQC (Andrews 2010) and Trim Galore (Krueger 2012; Figure S2). Following the removal of poor‐quality nucleotides and adapter sequences, we filtered out reads shorter than 21 nucleotides before proceeding with downstream analysis. Reads were then mapped to the zebra finch genome assembly (RefSeq bTaeGut1.4.pri) using Bowtie 2 (Langmead and Salzberg 2012; Rhie et al. 2021) set with default sensitive parameters for seeding and multiseed alignment. SAMtools (Li et al. 2009) was used to convert between SAM and BAM file formats, to filter out reads that did not uniquely map to the genome, and to generate corresponding index files. Sequence data files can be found as NCBI BioProject PRJNA1256487.

### 2.5 Peak Calling for Differential Analysis and Gene Prediction

Peaks were first called for each sample against input using MACS2 with an effective genome size of 2.09E + 09 and a *q* value cutoff of 0.01, considering peak consistency checks and IDR (Zhang et al. 2008; Li et al. 2011; Feng et al. 2012). The reads and peaks were assessed using ChIPQC, and metrics were compared with one‐way ANOVAs followed by post hoc Tukey‐HSD tests as appropriate (Carroll et al. 2014). To then identify putative regulatory regions that were differentially associated with H3K27ac based on age and sex, we compared males across age (P23M vs. P30M), females across age (P23F vs. P30F), and males and females at each age (P23M vs. P23F and P30M vs. P30F). Regions with differential H3K27ac occupancy were identified with DESeq2 core routines in DiffBind using default settings and full library size normalization (Stark and Brown 2011; Ross‐Innes et al. 2012; Love et al. 2014). Because H3K27ac ChIP‐seq can return peaks of varied lengths, differential regions were called both with and without utilizing the DiffBind summit parameter. Outputs were then merged using bedtools (Quinlan and Hall 2010; Quinlan 2014), maintaining broader peaks at locations of overlap. Regions with differential H3K27ac occupancy with an FDR < 0.05 were reported and used for gene prediction. ChIPseeker using NCBI genome annotation corresponding to the bTaeGut1.4.pri assembly (GCF_003957565.2) was used to annotate differential regions (1) to determine the distribution of peaks across genomic features and (2) to assign protein‐coding genes that may be regulated (Yu et al. 2015; Figure S2). Predicted gene names were entered into the Genotype‐Tissue Expression (GTEx) portal to check for human adult cortex expression (calling “present” those with >2 TPM in GTEx v10 on 08/18/25).

### 2.6 Peak Calling for TFBS Analysis

TFBSs are located within nucleosome‐free regions (NFRs), stretches of the genome closely flanked by, but not directly bound in, nucleosomes. As NFRs are the most probable location of TFBSs, and NFR identification reduces the amount of sequence utilized for TFBS identification, considering NFRs reduces the risk of false positives. To facilitate identification of NFRs, we called broad peaks with a *q* value cutoff value of 0.1, as NFRs are more accurately identified within broad peaks compared to narrow peaks and the peaks were further refined for NFR with HisTrader (Zhang et al. 2008; Feng et al. 2012). Profiles from biological replicates were merged using bedtools (Quinlan and Hall 2010; Quinlan 2014) to produce a single NFR profile for each sample. To identify TFBSs enriched within the NFRs of differential regions, we reduced the length of the regions to be analyzed by intersecting the original differential regions (peaks called by MACS2 at *q* value cutoff 0.01, as in the prior section) with the NFR regions and then converted region files into FASTA files for TFBS analysis (Quinlan and Hall 2010; Quinlan 2014). TFBS enrichment was implemented with Simple Enrichment Analysis (SEA) (Bailey et al. 2015; Bailey and Grant 2021), belonging to the MEME Suite of programs, with a background model generated by shuffling the input sequences and an *E* value ≤10 cutoff (Figure S2). The JASPAR CORE (2022) nonredundant vertebrates database (Castro‐Mondragon et al. 2022) was used as input for the TF motifs tested. TFs identified from enriched binding site analysis were compared to the GTEx portal to check for human adult cortex expression (calling “present” those with >2 TPM in GTEx v10 on 08/18/25).

### 2.7 GO Term Enrichment Analysis

GO term enrichment analysis was performed on TFs identified from binding site analysis and predicted gene sets using ShinyGO v0.80 (Ge et al. 2020). The human genome (GRCh38.p13) was utilized for GO term enrichment analyses with HGNC (HUGO Gene Nomenclature Committee) gene names. Background was composed of all protein‐coding genes in the genome (UCSC Table Browser v112). Many enriched TFBSs (as above, initially identified with SEA) were for protein dimers, but GO is not designed to accept protein dimers as input. In these cases, TFBS dimers were split into their component proteins when performing GO enrichment analysis. We focused on presenting results that had clear ties to the nervous system, though we recognized that categories reflect existing knowledge, so some TFBS or genes that subsequently become relevant may not be represented in figures. For TFBSs, we excluded GO terms directly related to mRNA transcription from figures. Tables of supporting information contain all results without reduction, cleaning, or alteration.

### 2.8 ChIP‐PCR Validation

Independent P23 and P30, male and female, auditory forebrain samples were dissected from normally reared birds for ChIP‐PCR validation (*n* = 12 individuals per age/sex to create *n* = 3 pools per group). ChIP for H3K27ac was performed as described in Cotney and Noonan (2015) using Protein A Dynabeads (Thermo Fisher, Cat# 10001D) and H3K27ac antibody (Active Motif, Cat# 39133) with samples sonicated to achieve fragments 200–500 bp (30% amp, 10‐s sonication with 10‐s rests for a total of 30 min; Model 120 Sonic Dismembrator; Fisher Scientific, Cat# FB120110).

Ten PCR primer sets were designed from the P30M‐over‐P30F differential peak analysis. An additional primer set was generated for a region around *MYH7B*, a gene with low brain expression. PCR reaction efficiencies were determined through a dilution curve analysis, and primer specificity was confirmed via melt curve analysis and further verified by gel electrophoresis (data not shown). The sequences of primers used are in Table 1.

**TABLE 1:** Primer sequences for ChIP‐PCR.

Peak (gene) name	Forward (5′–3′)	Reverse (5′–3′)
autosomal‐1 (CTIF)	GAATATGTGGGAGGGGAAGCC	AGCTATCTGGTGCCAACTTGT
autosomal‐2 (LOC121468138)	GTCTCCTTCCAGAAGTCCTCAG	ACTGGGGTGATACCAGGCTC
autosomal‐3 (PCGF3)	AACAGCGCTGAGGGGAAG	GGTACCTTGTGTGCTTTGGC
autosomal‐4 (MAP1B)	CTGGTCGCCGCTTTCAAATG	TGTCCTACTGCTGTTAGTCTGC
Autosomal‐5 (RASSF8)	CGAAGTCAGTCGTCACCCG	ACATTGCTGTTCGTCTGGGTT
Z chromosome‐1 (LOC115490768)	CGCTGTGCATCGTTGTTTGTA	GGGCAGTCACCAGCATGAAG
Z chromosome‐2 (PRICKLE1)	AGTGGTGCTTTGGTGGTACG	TCAGCCTGACATGGCAAACA
Z chromosome‐3 (STK3)	GGGAAGAAAGGTGGGATGGC	CCACTCTCCCCAGTATGCAA
Z chromosome‐4 (SPON1)	ATGAGCCCTACCCTGCCAAC	GCAGGTACAGGTCTCTCTGG
Z chromosome‐5 (SECISBP2)	GTCCATGAGCGTAACAGTGC	CAGGCATTAACTTCCAGTGAGC
GAPDH (positive, normalization)	CTACAGCTGCTGACGAAGAGT	GGTATGCGCTGCTTTCACCT
MYH7B (negative)	AGCTCTTGACCTTGCTCTGC	TGGGACTCTGCTAAAGGTGG

Real‐time PCR was performed on triplicate 20 µL reactions containing 0.046 µg of immunoprecipitated chromatin using SsoAdvanced Universal SYBR Green Supermix (Bio‐Rad, Cat# 1725274). The reactions were performed on a CFX384 Touch Real‐Time PCR machine (Cat# 1855484, Bio‐Rad) using the following cycling conditions: an initial denaturation and polymerase activation at 98°C for 3 min, followed by denaturation at 98°C for 15 s and annealing/extension at 60°C for 30 s for a total of 50 cycles (negative control reactions had Cq up to 41.4). For each biological sample (*n* = 3 per group), the average Cq value of the technical replicates was calculated for both the target primer set and GAPDH. The ΔCq value was then calculated by subtracting the average Cq value of GAPDH from the average Cq value of the primer set of interest (ΔCq = Cq_Primer_ − Cq_GAPDH_). Relative expression was quantified using the 2ΔCq method. One‐way ANOVA followed by post hoc Tukey‐HSD tests on the 2ΔCq values were used to identify potential statistical differences between the experimental groups, with a significance threshold set at *α* < 0.05.

### 2.9 Next‐Generation RNAseq and Analysis

To provide an RNA dataset to inform ChIP‐seq findings, auditory forebrain RNA was isolated with the RNeasy Micro Plus Kit (Qiagen, #74034) with RNase‐Free DNase I treatment (Qiagen, #79256). PolyA‐selected RNA Tecan libraries (Ovation Ultralow v2) were prepped and sequenced via NovaSeq 6000 with S4 flow cells to acquire 150 nt paired‐end reads by the Roy J. Carver Biotechnology Center at the University of Illinois, Urban‐Champaign. Reads were processed and quality‐checked using Partek Flow (Illumina). Reads were aligned with STAR (v2.7.8) to the zebra finch reference genome (bTaeGut1.4.pri). RNA abundance was quantified with library‐size normalized (counts per million [CPM]) data, and low abundance features were removed with the lowest average coverage threshold (1.0). Differential expression was evaluated in Partek Flow using one‐way ANOVA, followed by multiple testing correction with the Benjamini–Hochberg (step‐up) procedure. We compared across age (P23M‐P30M, P23F‐P30F) and sex (P23M‐P23F, P30M‐P30F). Statistically significant (*p* < 0.05) gene names were supplied as a single ranked list to GOrilla to assess functional enrichment categories using the human reference (Eden et al. 2009). Genes with final read counts >2 were cross‐referenced with lists of TFBS and predicted genes generated from ChIP‐seq analysis.

## 3 Results

### 3.1 ChIP‐seq Data Are High Quality

**FIGURE 1:**
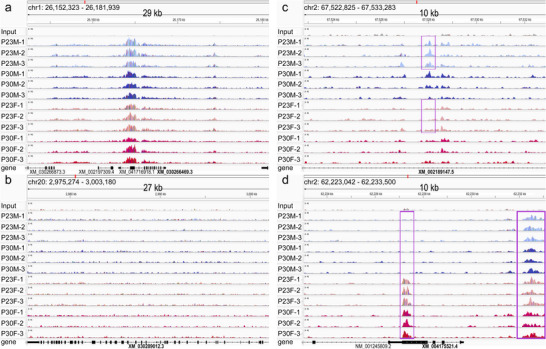
**Integrative genome viewer tracks showing peak profiles for all samples**. (a) H3K27ac signal is present in all samples near the 5′ end of *GAPDH* (XM_030266469.3), a ubiquitously expressed housekeeping enzyme (scale = 100). (b) The signal is universally low across *MYH7B* (XM_030289012.3), a gene expressed in liver but low in brain (scale = 30). (c) Peak (*JARID*; XM_002189147) shows increased H3K27ac signal in P23M compared to P23F (boxes; scale = 40). (d) One peak in *TUBB1* (XM_004175521.4) shows H3K27ac signal in females but not males (center box), in contrast to the region in the right‐side box, where signal is detected across all samples (scale = 40).

Sample quality and reproducibility measures indicate that the data were suitable for further analysis (Table 2, Figure 1, Table S1, Figure S3). An average of 34,271,218 reads were obtained per sample (minimum 28,988,336; Table 2, Table S1). The median quality score of bases across samples was 36, with no less than a score of 32 at any position (data not shown). Following read trimming, samples maintained an average of 33,182,950 reads (minimum = 28,135,662; Table S1). Quality metrics were similar across samples (Table 2, Table S1). One‐way ANOVAs revealed no main effect of group on reads removed (*F*(3,8) = 3.14, *p* = 0.087; range = 1.1%–6.2%), the proportion of reads mapped to the genome (*F*(3,8) = 0.926, *p* = 0.47; range = 94.99%–95.77%), proportion of reads in peaks (*F*(3,8) = 1.15, *p* = 0.39; range = 19.1%–26.2%), average peak length (*F*(3,8) = 0.496, *p* = 0.69; range = 656–698), or average peak signal value (*F*(3,8) = 1.083, *p* = 0.41; range = 4.1–4.9). Correlation values across individual replicates revealed that same‐sex and same‐age samples were more similar to each other than samples of the other age and sex; sample “P30F‐N1” demonstrated a lower correlation than all other samples, at 0.7 (Figure S3a). Before DiffBind analysis, there was a significant main effect (*F*(3,8) = 6.58, *p* = 0.015) of peak number, with post hoc Tukey‐HSD tests showing that P23M samples had a significantly greater number of peaks than P30M samples (*p* adj = 0.018; Table 2, Table S1).

**TABLE 2:** Summary of ChIP‐Seq quality metrics.

Sample	Read#	propRem	propAlign	Filt%	FragL	RelCC	SSD	FRiP	#Peaks
**P23M‐1**	39,795,925	0.02	0.95	8.09	204	2.23	0.80	23.2	58,078
**P23M‐2**	36,588,134	0.01	0.96	7.64	196	2.69	0.82	26.6	64,275
**P23M‐3**	37,026,827	0.02	0.95	7.27	204	2.82	0.78	25	60,853
**P23F‐1**	33,646,290	0.03	0.95	8.71	233	2.18	0.80	21.5	51,750
**P23F‐2**	35,061,341	0.03	0.96	7.71	203	3.02	0.80	23.3	53,407
**P23F‐3**	41,029,244	0.03	0.95	8.58	206	2.22	0.86	22.9	57,271
**P30M‐1**	31,103,728	0.03	0.95	8.44	229	2.29	0.78	21.8	49,380
**P30M‐2**	28,988,336	0.03	0.96	8.02	233	2.92	0.77	22.8	48,990
**P30M‐3**	31,454,411	0.05	0.96	8.17	198	2.19	0.78	23.3	55,352
**P30F‐1**	33,120,652	0.07	0.95	9.21	200	1.88	0.77	19.1	48,942
**P30F‐2**	30,301,607	0.06	0.96	7.79	230	3.2	0.81	25.4	54,198
**P30F‐3**	32,162,950	0.03	0.95	8.06	232	3.02	0.81	23.1	52,093

*Note*: Read# = number of reads obtained. propRem = proportion of reads removed after filtering and trimming. propAlign = the proportion of reads aligning to the genome assembly. Filt% = percentage of reads removed from downstream analysis for not meeting the *q* value cutoff threshold of 15. FragL = estimated mean fragment length determined by systematically shifting the reads on each strand toward each other until the minimum genome coverage is achieved. RelCC is obtained by comparing the maximum cross‐coverage peak to the cross‐coverage at a shift size corresponding to the read length. Higher scores (generally 1 or greater) indicate good enrichment. SSD (standardized standard deviation) is computed by looking at the standard deviation of signal pile‐up along the genome normalized to the total number of reads. #Peaks = total number of peaks called for each sample.

Abbreviation: FRiP = fraction of reads in peaks.

Consistent with expected profiles, peaks were predominately located in promoter, intronic, and distal intergenic regions for all conditions (Figure 1, Figure S3b). We assessed the number of putative proximal promoters and enhancers using the definition that promoters were ≤5 kb upstream of transcriptional start sites (TSS). Putative enhancers here were the remainder of the H3K27ac‐defined peaks. This definition may be a conservative estimate of enhancers because some can be more proximal to TSS, though H3K27ac‐marked regions have also been related to several *cis*‐regulatory element categories (Creyghton et al. 2010; Huang and Ovcharenko 2022; Kim and Wysocka 2023). One‐way ANOVA (*α* < 0.05) did not find a significant main effect in the numbers of putative promoters (*F*(3,11) = 3.22, *p* = 0.08) but a trend toward a difference in the number of putative enhancers (*F*(3,11) = 3.93, *p* = 0.05; Figure S3c,d). We confirmed that HisTrader refined broad peaks for NFR and TFBS enrichment analysis (Figure S3e). Overall, 96% of the putative TFs are expressed in adult human cortex, as are 92% of the predicted genes (data not shown).

Finally, we performed ChIP‐PCR based on 10 peaks from the P30M‐over‐P30F comparison and one region near a gene with low brain expression (MYH7B, based on RNAseq data and GTEx Portal accessed 8/18/2025), using biologically independent samples (Figure S3f–p). After normalization to GAPDH, 7 of the 10 peaks confirmed noticeably greater signal in the P30M compared to P30F samples, with 2 reactions showing statistical differences (chrZ‐1 (LOC115490768): *p* < 0.002; chrZ‐5 (SECISBP2): *p* < 0.0004). We also included P23 samples. Amplification for chrZ‐1 (LOC115490768) revealed that it was also significantly enriched in P23M compared to P23F auditory forebrains (*p* < 0.04), with two other regions (chrZ‐3 (STK3) and chrZ‐5 (SECISBP2)) also showing reliable, though not significant, differences in P23M and P23F.

### 3.2 Comparison of P23 and P30 Male H2K27ac Peaks Revealed the Potential for Select TF to Regulate Biological Functions Related to Sensitive Period Opening

**FIGURE 2:**
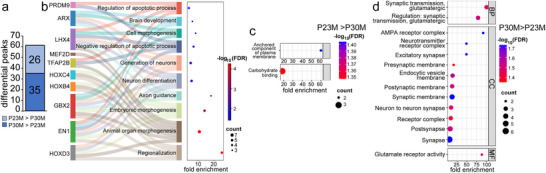
**Differences between P23 and P30 male auditory forebrain profiles**. (a) The number of P23M‐over‐P30M and P30M‐over‐P23M differential H3K27ac peaks. (b) Sankey dot plot from GO Biological Process enrichment analysis of P30M‐over‐P23M TFs. (c and d) Dot plots of GO term enrichment analysis performed on predicted genes from (c) P23M‐over‐P30M, and (d) P30M‐over‐P23M differential region analysis. BP = biological process; CC = cellular component; MF = molecular function.

To identify novel TFs and genes that may be differentially acting in P23 and P30 male auditory forebrains, we first performed a direct comparison of peak sets to identify those with different H3K27ac occupancy. We found a total of 61 statistically differential H3K27ac‐defined regions between P23 and P30 males, with roughly equivalent numbers of “P30M‐over‐P23M” and “P23M‐over‐P30M” peaks (Figure 2a, Table S2).

We then identified the TFBSs enriched in these differential regions. P23M‐over‐P30M regions were enriched with TFBSs for a single TF, MEF2A, and P30M‐over‐P23M regions were enriched with TFBSs for 13 TFs, including MEF2D (Figure S4a, Table S3). GO term analysis of P30M‐over‐P23M TF motifs highlighted general themes of cellular proliferation, differentiation, and tissue development but also highlighted themes specifically relevant to brain development, including neurogenesis (ARX, LHX4, HOXD3, EN1, GBX2), neuronal differentiation (ARX, LHX4, HOXD3, EN1, GBX2), apoptosis (TFAP2B, EN1), regionalization (HOXD3, EN1, GBX2, HOXB4, HOXC4), and projection guidance (ARX, LHX4, GBX2; Figure 2b, Table S4).

To further investigate the potential functional differences between P23 and P30 male auditory forebrains, we annotated each H3K27ac‐based differential region to a protein‐coding gene and performed GO functional analysis (Tables S2 and S5). The only categories significantly enriched in the genes associated with P23M‐over‐P30M regions were anchored component of plasma membrane and carbohydrate binding (Figure 2c, Table S5). Terms significantly enriched in the genes associated with P30M‐over‐P23M regions highlight processes related to synaptic function, with an emphasis on glutamatergic signaling (e.g., *CACNG3, SYT1, GRM4, GRIA3, KCTD16*; Figure 2d, Table S5).

### 3.3 Very Few Regulatory Regions Differed Between P23 and P30 Female Auditory Forebrains, but Analysis Still Revealed Biological Processes

**FIGURE 3:**
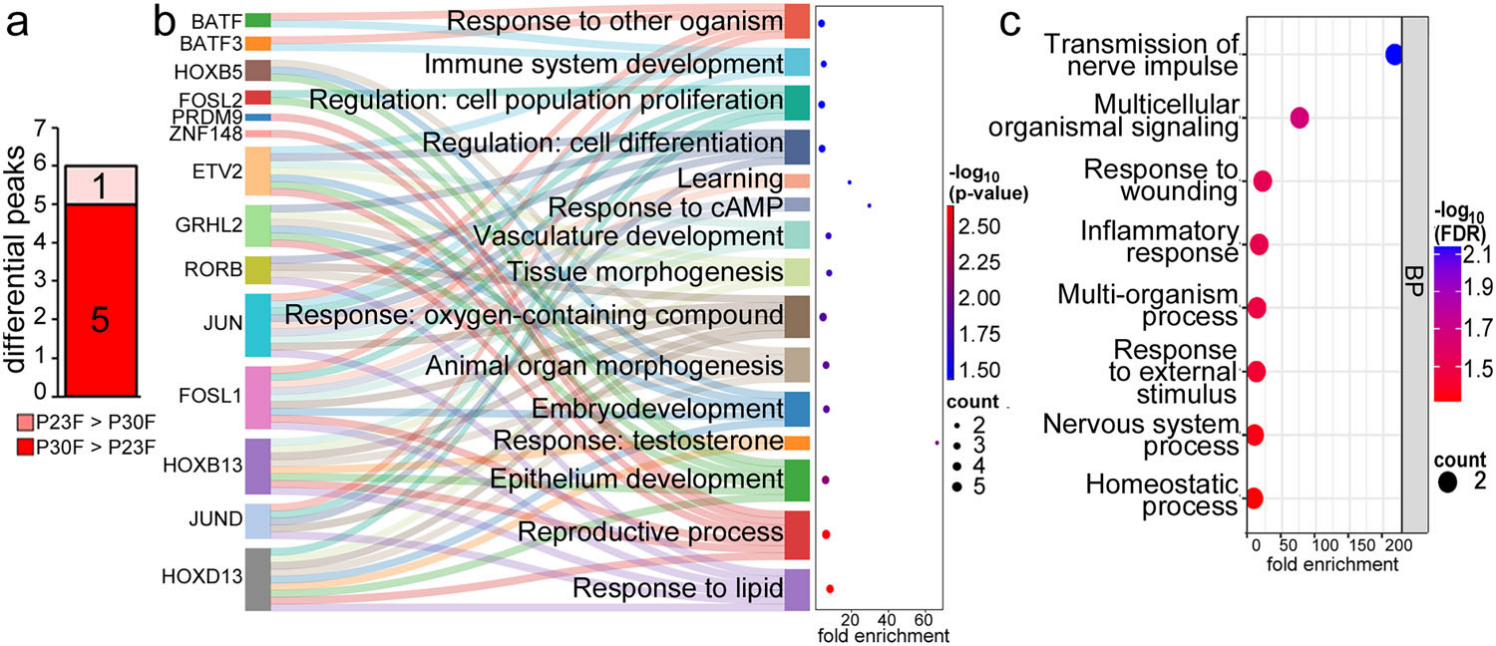
**Differences between P23 and P30 female auditory forebrain profiles**. (a) The number of P23F‐over‐P30F and P30F‐over‐P23F peaks with differential H3K27ac signal. (b) Sankey dot plot from GO biological process enrichment analysis of P30F‐over‐P23F TFs. (c) Dot plot displaying GO results from genes predicted from the P30F‐over‐P23F differential regions. BP = biological process.

Statistical analysis returned only 6 significant regions with differential H3K27ac signal between P23 and P30 female auditory forebrains (Figure 3a, Table S2). Five of the regions were more accessible in P30 female samples (P30F‐over‐P23F), and one of the regions was more accessible in P23 female samples (P23F‐over‐P30F).

We identified TFBSs enriched in the regions with differential H3K27ac signal between P23 and P30 female auditory forebrains. It was not possible to perform TF motif enrichment analysis for the one P23F‐over‐P30F peak, but P30F‐over‐P23F regions were enriched with binding sites for 14 TFs (Figure S4b, Table S3). Despite the low numbers, GO term analysis of P30F‐over‐P23F TFBSs highlighted general themes of cellular proliferation (e.g., FOSL2, HOXD13, JUND, FOSL1, and JUN) and differentiation (e.g., ETV2, BATF3, BATF, and JUN), tissue development (e.g., FOSL2, GRHL2, ETV2, HOXB5, HOXD13, and HOXB13), and responsivity (e.g., HOXD13, JUND, HOXB13, FOSL1, JUN, and RORB; Figure 3b, Table S3).

The single P23‐over‐P30 female region was assigned to the gene *CCDC43*, a broadly expressed cytosolic protein that has predominantly been investigated for its potential role in cancer cell growth and metastasis (Table S2; Wang et al. 2020; Chen et al. 2022). The three characterized genes associated with P30F‐over‐P23F regions were *DRD5*, *JAM3*, and *BTF3* (*LOC116806881*). *DRD5* encodes a G‐protein coupled dopamine receptor shown to regulate hippocampal‐dependent memory formation (Sariñana et al. 2014; Sarinana and Tonegawa 2016). *JAM3*, a gene encoding a junctional adhesion molecule crucial for neuron development and cell adhesion, was also associated with genomic regions exhibiting greater H3K27ac signal in P30 versus P23 male zebra finches (Table S2; Mochida et al. 2010). However, the differential male regions did not overlap with any regions in females, indicating the potential for distinct regulation of this gene. *BTF3* encodes basic TF 3, a protein that complexes with RNA polymerase II to initiate transcription (Zheng et al. 1987). *JAM3* and *DRD5* alone supported several significantly overrepresented GO categories, including “transmission of nerve impulse” (Figure 3c, Table S5).

### 3.4 Sexually Dimorphic H3K27ac Peaks Were Predominately Localized to Sex Chromosomes

**FIGURE 4:**
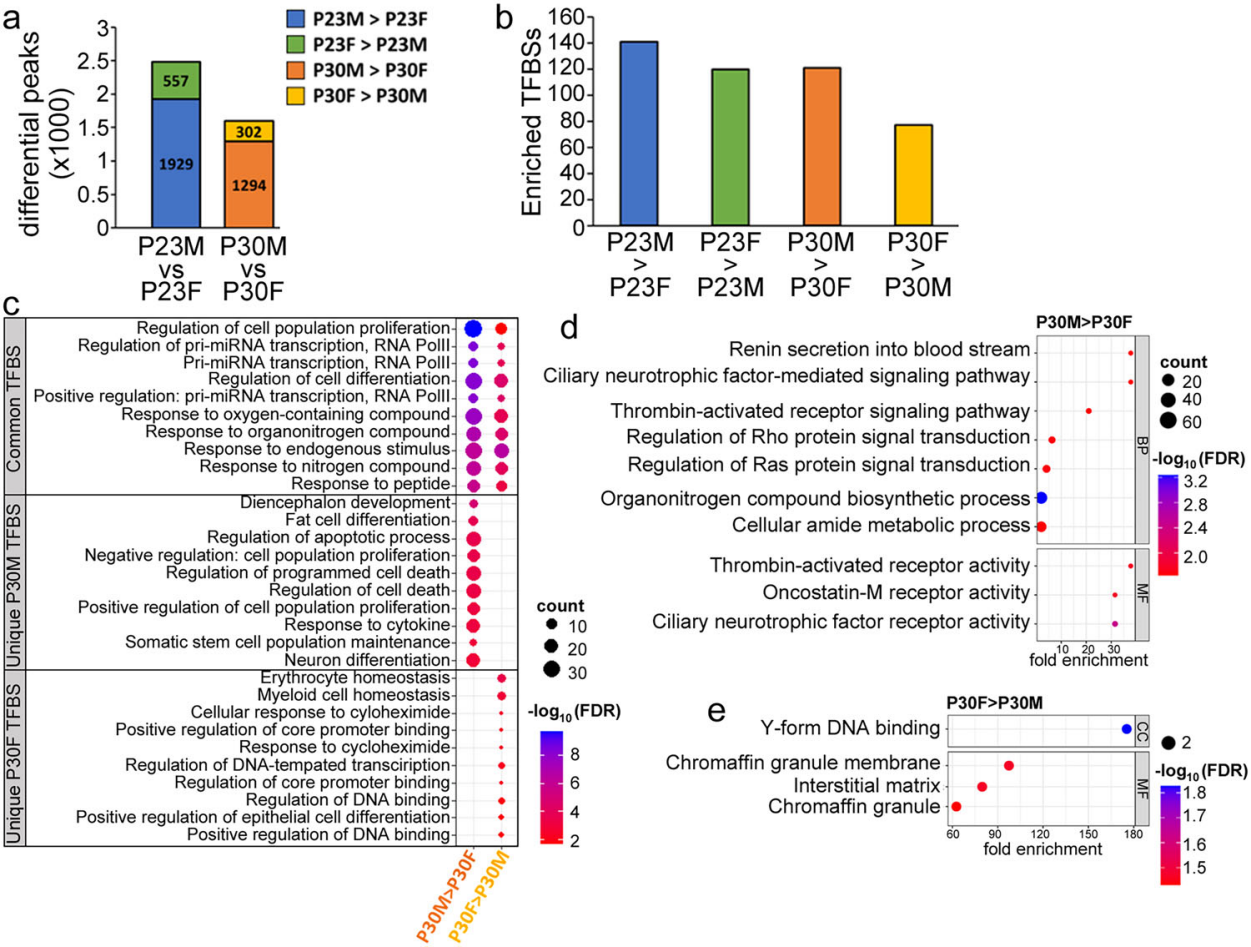
Direct sex comparisons at P23 and P30, with GO enrichment at P30. (a) The number of sexually dimorphic peaks associated with H3K27ac at each age. (b) The number of TF motifs enriched in sexually dimorphic regions. (c) Dot plot comparing the “common” (enriched in male‐over‐female and female‐over‐male analyses) and sex‐unique results from Biological Process GO analysis of P30 TFBSs; a subset of categories with the lowest enrichment FDR values are shown. (d and e) Dot plots displaying the results from GO analysis performed on genes predicted from the (d) P30M‐over‐P30F regions or (e) P30F‐over‐P30M regions. BP = biological process; CC = cellular component; MF = molecular function.

We directly compared H3K27ac‐defined peaks between age‐matched male and female auditory forebrains (Figure 4a, Table S2). Overall, 80.1% and 94.2% of the sexually dimorphic regions were localized to sex chromosomes at P23 and P30, respectively (Table S2). As expected, all sexually dimorphic regions localized to chromosome W provided greater H3K27ac signal in females than males (male zebra finches have a ZZ sex chromosome complement, whereas females have one Z and one W chromosome). With the exception of one region, the sexually dimorphic regulatory regions on the W chromosome were observed at both P23 and P30. All sexually dimorphic regions located on chromosome Z returned more H3K27ac signal in males than females (Table S2).

### 3.5 Analysis of Overrepresented TFBSs Revealed Development, Responsivity and Signaling, and Immune System Processes May Be Sexually Dimorphic at P23 and P30

We determined the TFBSs that were enriched within the sexually dimorphic regions and identified between 78 and 149 TF motifs across the four comparisons (P23M‐over‐P23F, P23F‐over‐P23M, P30M‐over‐P30F, and P30F‐over‐P30M; Figure 4a,b, Table S3). GO analysis of biological process categories highlighted significant overrepresentation of processes related to the immune system, signaling and responses, and developmental processes (Table S4).

### 3.6 GO Analysis Indicates Sex Differences That May Relate to Distinct Learning Mechanisms at P30

We then focused on the enriched TFBSs and predicted genes associated with sexually dimorphic regulatory regions at P30 because behavioral evidence demonstrates both males and females successfully perform sensory song learning at this age. Therefore, differences observed here may be the most directly related to sex differences in auditory forebrain organization as it pertains to the ability to learn. GO analysis using the TFBS data revealed a subset of biological processes that were overrepresented in both male‐over‐female and female‐over‐male comparisons (“common”; Figure 4c, Table S4). These include processes related to cellular development, responses, and RNA polymerase III. There were also themes unique to either the P30 male‐over‐female or P30 female‐over‐male TF motif data (Figure 4c). For example, categories of cellular differentiation, proliferation, and survival were exclusive to the male‐over‐female data, whereas processes of transcriptional regulation, homeostasis, and specific responses were represented in the female‐over‐male dataset.

GO analysis performed on the genes associated with P30 sexually dimorphic H3K27ac‐defined regions revealed no overlap between male‐over‐female and female‐over‐male terms (Tables S2 and S5). Male results include categories related to signaling, including Rho/Ras signaling and ciliary neurotrophic factor receptor (*CNTFR*) signaling (Figure 4d). Female categories, based on two predicted genes, suggest regulation of the interstitial matrix and chromaffin granules, which are catecholamine‐releasing cells in the adrenal gland (Figure 4e).

### 3.7 Cross‐Referencing the Four Sexually Dimorphic Profiles Identified “Core” and Sex‐Specific TF Motif Sets

**FIGURE 5:**
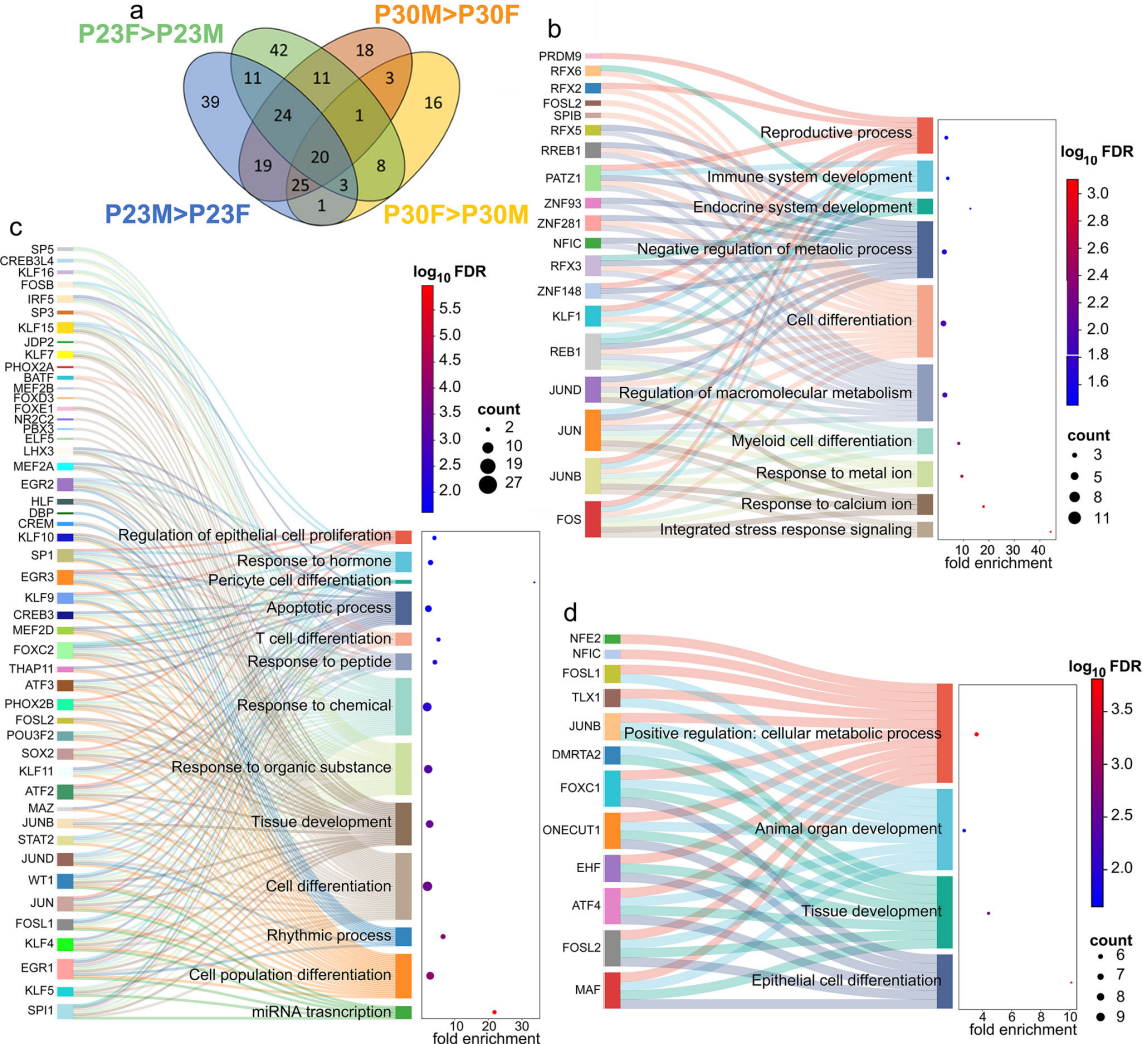
TFBSs enriched in sexually dimorphic regions that are cross‐referenced reveal core, sex, and age selective TF motifs and functions. (a) Venn diagram representing the number of enriched TFBSs from the four sexually dimorphic pairwise comparisons. (b–d) Sankey dot plot displaying results from biological process GO term enrichment analysis performed on (b) core TFBSs, (c) male shared TFBSs, and (d) female shared TFBSs.

To examine any shared enriched TFBSs that might provide functional insight, we cross‐referenced the four TF motif lists (P23 male‐over‐female, P30 male‐over‐female, P23 female‐over‐male, P30 female‐over‐male). This process showed that 114 TFBSs (24.7% of the total 462 TFBSs) were unique to a single comparison, 53 (11.5%) were present in two comparisons, 54 (11.7%) TFBSs were shared across three comparisons, and 20 (4.3%) TFBSs were present in all four comparisons (Figure 5a, Tables S3 and S6).

### 3.8 The Set of “Core” TF Motifs Highlights Developmental Plasticity Processes

The cross‐referencing analysis revealed a “core” subset of 20 TFs that had binding sites enriched in all four groups that is independent of age and sex (Figure 5a, Figure S5, Tables S3 and S6). Core TFBSs included immediate early genes (IEGs) prominently involved in learning and memory formation such as CREB1 and AP‐1 TF complex members JUN, JUNB, JUND, FOS, FOSL1, and FOSL2. Additionally, the core set contained TFBSs that regulate brain development, including SP4, ZNF148, ZNF281, and RFX4 (Table S6). GO biological process analysis demonstrated that the TFs enriched with motifs in the core group are overrepresented in categories related to cellular responses, including those related to cognition and learning and memory, as well as aging and cellular differentiation (Figure 5b, Table S4).

### 3.9 Sex‐Specific TF Motifs Highlighted Developmental Plasticity Processes

The cross‐referencing analysis also revealed the overrepresented TF motifs that were shared within sexually dimorphic regions in both P23 and P30 auditory forebrains. Shared male TF motifs were those that had enriched TFBSs in both P23 and P30 male‐over‐female comparisons, and the shared female TFBSs were enriched in differential peaks of both P23 and P30 female‐over‐male comparisons. Excluding the core TF motifs already examined, 19 TFBSs are present only in the male‐over‐female comparisons, and 8 TFBSs are present only in the female‐over‐male datasets (Figure S6, Tables S2 and S6). Male‐exclusive TF motifs included IEGs such as EGR‐1 and JUN dimers, and NRF1. The JUNB::FOSL1 dimer was overrepresented in the female‐exclusive TFBS list, as were ONECUT1 and DUX. Biological process GO terms overrepresented in the male‐exclusive TFBSs generally revealed coordination of cellular proliferation and survival, myelination, and responsivity (Figure 5c, Tables S4 and S6). The same analysis based on the female‐exclusive TFBSs returned categories related to development and metabolism (Figure 5d, Tables S4 and S6).

### 3.10 Cross‐Referencing TF Motif Profiles Across Sex Revealed That a Similar Proportion Were Enriched in Both Male‐ and Female‐Biased H3K27ac‐Defined Regions at P23 and P30

When we assessed the overlap in sexually dimorphic profiles, we found that at P23, 59 TFBSs (41.3% and 48.8% of male‐over‐female and female‐over‐male TFBSs, respectively) were overrepresented in both male‐over‐female and female‐over‐male regions. At P30, the number of shared TFBSs is 49 (40.2% and 63.6% of male‐over‐female and female‐over‐male TFBSs, respectively; Tables S3 and S6).

After excluding the core TF motifs that were already examined, we performed GO analysis on TF motifs that were shared between sexes. Results showed almost exclusive enrichment in biological process terms directly related to transcription at both ages (Tables S4 and S6). However, at P23, categories of brain and tissue development, including those of cellular differentiation, proliferation, and survival, were also overrepresented, as were those specifically for miRNA transcription. Categories that emerged from the P30 TF motif list also included those related to cell migration and responsivity (Table S4).

### 3.11 RNAseq Confirms Expression of a Majority of Predicted TFs and Genes in the P23 and P30 Auditory Forebrain

We compared the enriched TFBSs and genes predicted from the H3K27ac peak analyses to RNAseq data we collected from an independent set of P23 and P30, male and female, auditory forebrains (Tables S7 and S8). mRNAs for 42% of the putative TFs and 66.5% of the predicted genes are detectable in our RNAseq data (Table S8). At P23, 23% of the P23M‐over‐P23F genes were localized to the Z chromosome; at P30, this proportion rises to 68%.

Analysis of the RNAseq data alone reveals signatures of processes represented in the H3K27ac data, including brain development and synaptic regulation (Tables S8 and S9). GO analysis of differentially abundant RNAs revealed several categories overrepresented at FDR <0.05, including the extracellular region, protein transport, G protein‐coupled receptors, and apoptotic mechanisms.

## 4 Discussion

Generally, neural organization emerges from the generation and survival of cells, their differentiation, their relative numbers and specific properties, and how they connect. These processes change across maturation and are influenced by experience; convergence can lead to sensitive periods, ages at which specific experiences have maximal effect on brain and behavior. Both male and female zebra finches perform adult behaviors that reflect juvenile sensory song learning, with a known critical period in males (Miller 1979a; Eales 1985, 1987; Morrison and Nottebohm 1993; Slater et al. 1993; Riebel 2000, 2003a, 2009; Riebel et al. 2002; Lauay et al. 2004; Roper and Zann 2006; Terpstra et al. 2006; Braaten 2010; Holveck and Riebel 2014; Chen et al. 2017; Diez et al. 2019). However, we still have very little insight into factors that may contribute to the onset of the male sensitive period and essentially no mechanistic data on juvenile females (Clayton 1988; Riebel 2000, 2009; Bailey and Wade 2003; Derégnaucourt et al. 2013; Holveck and Riebel 2014; Ahmadiantehrani and London 2017; Kelly et al. 2018). Understanding the baseline neural properties that support experience‐dependent plasticity at different ages has potentially wide‐reaching implications for typical and atypical development. Despite the short developmental span investigated—just 1 week—our analysis revealed genomic regions and potential TFs and genes that may contribute to the ability to learn and added to evidence that the developing auditory forebrain is sexually dimorphic.

Prior work has demonstrated a behavioral and molecular transition into the critical period for sensory song learning between P23 and P30 in males (Roper and Zann 2006; Ahmadiantehrani and London 2017). Although only 1 week separates our birds, we did identify putative *cis*‐regulatory elements based on H3K27ac peaks that were enriched with binding sites for TFs implicated in brain development and organization. Some identified TFs may have multiple effects related to developmental learning onset. For example, two MEF2 (myocyte enhancer factor 2) TFs emerged from the P23 to P30 male analyses that were confirmed by RNAseq analysis. MEF2 proteins can operate as transcriptional repressors or activators and can switch between states as a result of activity (Gaudilliere et al. 2002; Shalizi and Bonni 2005; Flavell et al. 2006; Shalizi et al. 2006; Potthoff and Olson 2007; Pulipparacharuvil et al. 2008; Assali et al. 2019). MEF2 TFs regulate neuronal survival, differentiation, and migration, as well as axon and dendrite features that effectively regulate synapses, including connectivity and elimination (Assali et al. 2019). Together, the MEF2 and other identified TF are positioned to direct instrumental aspects of maturational programming that could give rise to a brain state capable of responding to experience.

Consistent with the potential for H3K27ac‐associated regions to be enhancers that determine cell subtypes, we identified enriched binding sites for TFs implicated in select subtypes (Serfling et al. 1985; Bulger and Groudine 2010; Creyghton et al. 2010; Calo and Wysocka 2013). For example, ARX and TFAP2B regulate GABAergic cells (Kitamura et al. 2002; Kato and Dobyns 2005). Inhibitory/excitatory balance and GABAergic cells, in particular, are central to cortical maturation and critical periods in primary sensory cortices (Hensch et al. 1998; Wehr and Zador 2003; Knudsen 2004; Hensch 2005; Takesian and Hensch 2013; Davis et al. 2015; Werker and Hensch 2015; Wood et al. 2017). GABAergic neurons are abundant in the auditory forebrain and contribute to genomic song response in adulthood, yet normally reared (as were those used for ChIPseq analysis) P25 and P45 males do not demonstrate significant differences in *GAD65*‐expressing cell densities within the higher order regions of the auditory forebrain responsible for sensory song learning (Pinaud et al. 2006, 2004; Pinaud and Mello 2007; Gogola et al. 2019). The role of these TFs in the transition to the critical period for male sensory song learning has not been examined and would benefit from further empirical testing.

Unlike the more commonly studied areas necessary for learned song production males, the auditory forebrain does not have macroscopic sex differences. It was an open question if juvenile females and males use the same mechanisms to become capable of sensory song learning and the extent to which mechanistic profiles may lend support for the presence of a sensitive period in females. Our “core” analysis did find evidence that broad processes such as development and cell responsivity were regulated between P23 and P30 females, as they were in males. Some TFBSs that were predicted from P23F compared to P30F peaks were identified from the within‐male analysis, too. These included those that regulate brain development, as well as AP‐1 and IEG TFs that have prominent roles in learning and memory formation. However, because the peaks identified in the female comparison did not overlap with those from the male comparison, these TFs would be binding to distinct regions of genome, likely leading to sex differences in their biological influence.

Similarly, direct sex comparisons highlighted distinct properties. For example, ciliary neurotrophic factor (CNTF)‐mediated signaling pathway and regulation of Rho and Ras signal transduction are overrepresented categories based on P30 male‐specific TFBSs. CNTF functions as a survival factor for nervous system cells and operates within sensory neurons to influence the developmental sex‐specific behaviors during postnatal brain maturation (Morris et al. 2004; Koemeter‐Cox et al. 2014; Jia et al. 2019). Ras and Rho are interconnected GTPase‐dependent molecular cascades that are critically involved in almost all aspects of neurodevelopment, including neurogenesis, neuron differentiation, vesicular trafficking, and synaptic plasticity (Chang et al. 2003; Govek et al. 2005; Ye and Carew 2010; Liang et al. 2019; Soriano et al. 2021). Two major downstream effector pathways of Ras and Rho are the ERK and mTOR cascades, both of which are necessary for juvenile male sensory song learning (Bailey and Wade 2003; Cheng and Clayton 2004; London and Clayton 2008; Ahmadiantehrani and London 2017). Genes annotated from P30 male‐over‐female peaks revealed a signature of synaptic plasticity involved in learning and memory, neurodevelopmental processes, and cognitive development (Pekhletski et al. 1996; Gerlai et al. 1998; Burgess et al. 1999; Payne 2008; Wu et al. 2014; Bornschein and Schmidt 2019; Chang et al. 1998; Lepicard et al. 2006; Wu et al. 2007; Wang 2008; Vigil et al. 2017; Zuo et al. 2019; Trivisano et al. 2020). Overall, it is thus plausible that the TFs and genes described here could alter the composition and function of the cellular auditory forebrain circuit distinctly in males and females during developmental learning, despite both sexes successfully learning from song exposure.

Our results do come with some caveats. Technically, TFBS prediction is just that, and the complicated biological process of protein binding to specific stretches of genomic DNA is not fully captured in sequence motif analysis (Badis et al. 2009; Gordân et al. 2009; Morris et al. 2011; Zhao and Stormo 2011). Further, although progress is being made in validating the function of *cis*‐regulatory elements, it is well known that different peak calling algorithms return distinct sets of differential regions, that motifs are often similar across different TFs especially within families, and that we do still assume the parameters for annotating regulatory regions with transcribed elements. These assumptions undoubtedly simplify the conformational relationships that exist across the genome (Ma et al. 2018; Kim and Shendure 2019; Vierstra et al. 2020; Spiegel et al. 2021; Pop et al. 2023; Portillo‐Ledesma et al. 2024). We recognize some of our findings are not obviously directly related to brain or development, and it is currently unclear if this reflects technical or knowledge limitations. Because of the small size of the auditory forebrain, we pooled individuals to create samples for bulk sequencing. The brain also includes multiple cell types that are assayed together in our bulk sequencing approach. However, we included three biologically independent samples, with consideration of genetic relatedness, and used animals from a genetically heterogeneous population. Given the biological heterogeneity of birds and tissue, results therefore must be sufficiently robust to emerge from that variation and thus serve to increase our confidence in outcomes.

The discoveries reported here provide the foundation for future work to test the role of specific TFs, genes, and processes for maturational and experience‐dependent contributions to neural organization and function. For example, it would be valuable to gain additional resolution of developing auditory forebrain at the single‐cell level, perform additional comparisons of these data to the rapidly expanding datasets of epigenetic and transcriptomic data within zebra finch and other species, leverage biological validations of enhancer sequences, and perform functional tests of the TFs and genes predicted from H3K27ac‐defined peaks.

## 5 Conclusion

For now, our data are consistent with prior molecular and behavioral evidence that suggested sex differences in how and when juvenile zebra finches might learn from tutor experiences. Our focus on putative regulatory regions allowed identification of possible TFs implicated in the transition to developmental learning. Because of TFs function to coordinate transcription of gene suites, they provide particular insight into processes that may be regulated to support or limit sensory song learning and those that are shared and distinct across males and females. We assessed the H3K27ac ChIP‐seq data in several ways to provide a comprehensive perspective and increase confidence in the results. We often had to extrapolate functional information for the TFs and genes predicted here because there are few studies specific to brain development and learning, especially in zebra finches. However, this investigation uncovered intriguing clues about specific processes, presenting novel targets for continued investigation into factors that may be essential to the overall organization and function of the auditory forebrain, and those that may be required for juvenile learning.

## Funding

This work was supported by Grants R56 NS110951 and R01 NS128286.

## Ethics Statement

All procedures were conducted in accordance with the NIH guidelines for the care and use of animals for experimentation and were approved by the University of Chicago Institutional Animal Care and Use Committee (ACUP no. 72220).

## Conflicts of Interest

The authors declare no conflicts of interest.

## Supporting information




**Supplementary Material**:dneu23017‐sup‐0001‐FigureS1‐S6.pdf

**Supplementary Material**:dneu23017‐sup‐0002‐TableS1.xlsx

**Supplementary Material**:dneu23017‐sup‐0003‐TableS2.xlsx

**Supplementary Material**:dneu23017‐sup‐0004‐TableS3.xlsx

**Supplementary Material**:dneu23017‐sup‐0004‐TableS3.xlsx

**Supplementary Material**:dneu23017‐sup‐0006‐TableS5.xlsx

**Supplementary Material**:dneu23017‐sup‐0007‐TableS6.xlsx

**Supplementary Material**:dneu23017‐sup‐0008‐TableS7.xlsx

**Supplementary Material**:dneu23017‐sup‐0009‐TableS8.xlsx

**Supplementary Material**:dneu23017‐sup‐0010‐TableS9.xlsx

## Data Availability

Sequence data files can be found as NCBI BioProject PRJNA1256487.
