# Supplementary material for: Chromatin Profiling Reveals Distinct Male and Female Trajectories for Developmental Learning Potential
Source: Dev Neurobiol. 2025 Nov 10;86(1):e23017. doi: 10.1002/dneu.23017 (PMC12603347; doi:10.1002/dneu.23017)
Supplement: Supplementary file 1 — Supplementary Material:dneu23017‐sup‐0001‐FigureS1‐S6.pdf [file DNEU-86-0-s001.pdf]

Supplementary Figures.

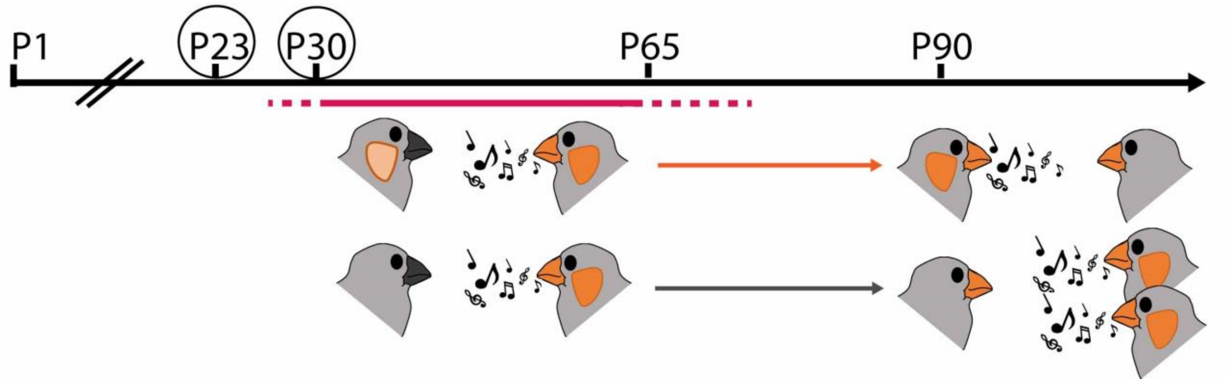

Supplementary Figure 1. Developmental timeline with behavioral significance and ages of collection. Top shows basic timeline of posthatch (P) development, starting at hatch (P1) and ending with adulthood (P90). Circles designate ages used in this study. Juvenile males (dark beak, light orange cheek patch) and females (dark beak, no cheek patch) are influenced by adult male (orange beak and cheek patches, music notes) tutor song experience between P30-65 (red bar) though ages may be extended for females (dashed red bar). Juvenile learning influences adult male song structure and adult female (orange beak, no cheek patch) song preference.

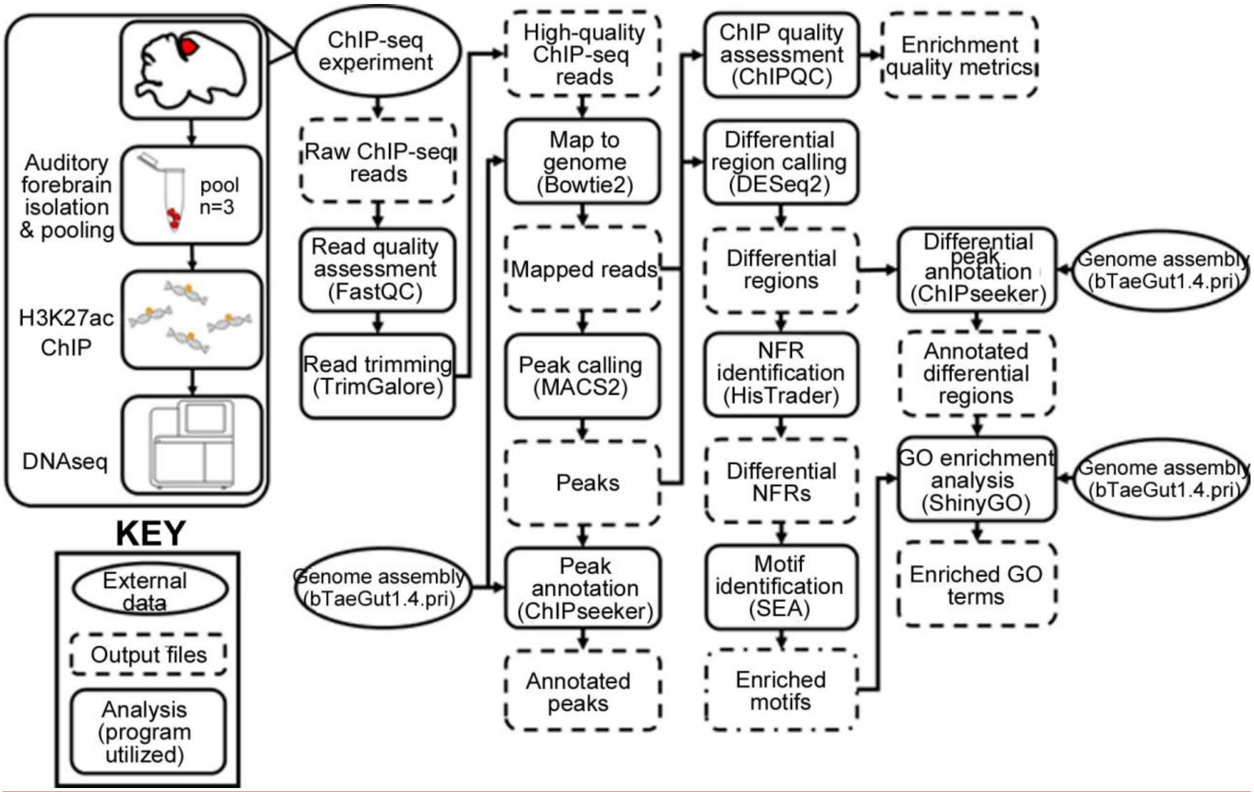

Supplementary Figure 2. Pipeline of methods including bioinformatic analysis pipeline. Pipeline steps and algorithms are depicted in solid boxes, with processed data types presented in dashed boxes between steps and externally-obtained data in solid ovals. Arrows indicate the directional flow of analyses.

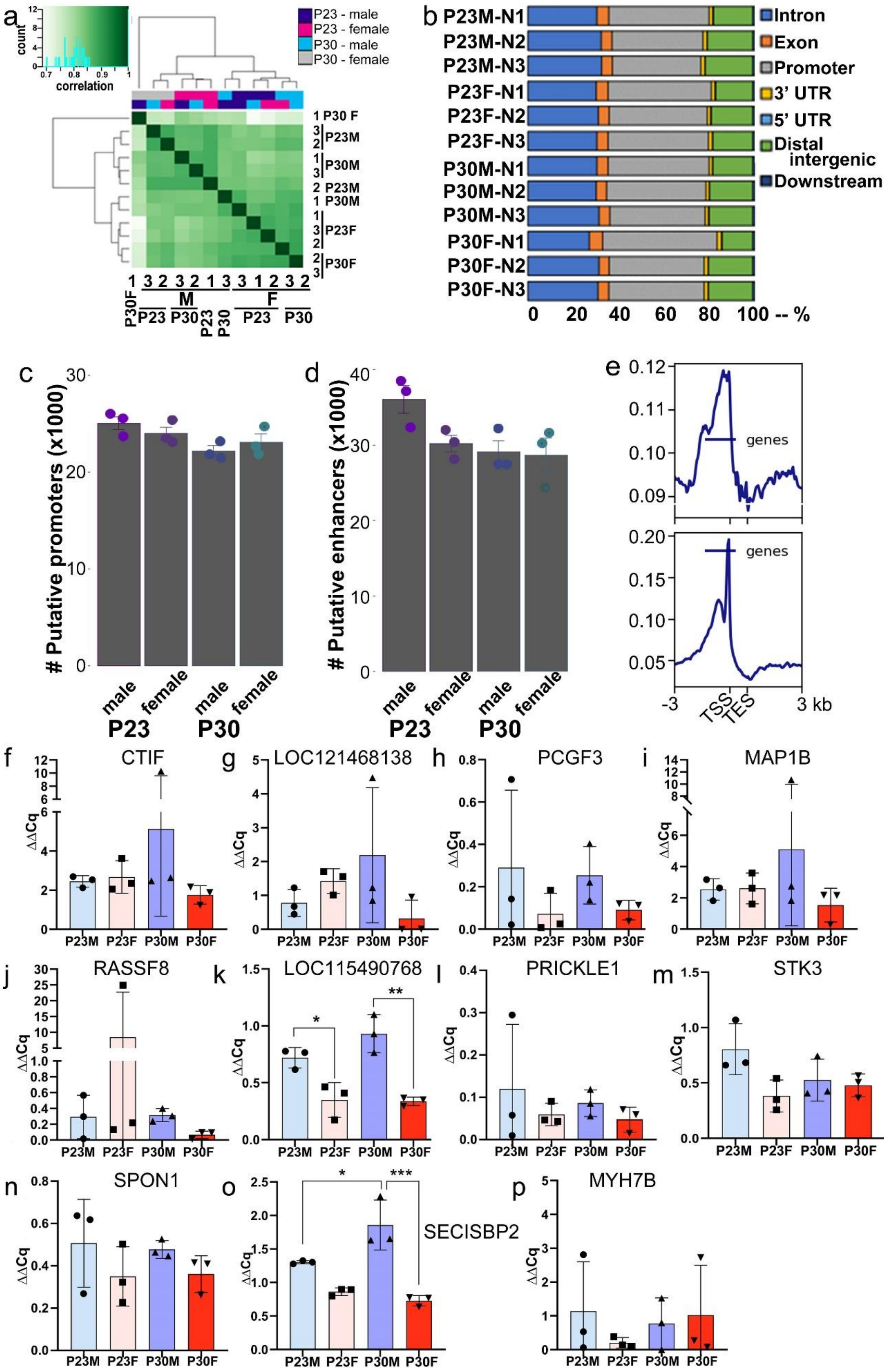

a
count
correlation
P23 - male
P23 - female
P30 - male
P30 - female
1 P30 F
P23M
P30M
2 P23M
1 P30M
P23F
P30F
M
F
P30F
P23
P30
b
P23M-N1
P23M-N2
P23M-N3
P23F-N1
P23F-N2
P23F-N3
P30M-N1
P30M-N2
P30M-N3
P30F-N1
P30F-N2
P30F-N3
Intron
Exon
Promoter
3' UTR
5' UTR
Distal intergenic
Downstream
0 20 40 60 80 100 -- %
c
Putative promoters (x1000)
male
female
P23
P30
d
Putative enhancers (x1000)
e
genes
-3
TSS
TES
3 kb
f
CTIF
ΔΔCq
P23M
P23F
P30M
P30F
g
LOC121468138
h
PCGF3
i
MAP1B
j
RASSF8
k
LOC115490768
*
**
l
PRICKLE1
m
STK3
n
SPON1
o
SECISBP2
*
***
p
MYH7B

Supplementary Figure 3. Analysis describing and demonstrating data quality. (**a**) Distribution of peaks across genomic features for each group. (**b**) Number of putative proximal promoters identified. (**c**) Number of putative enhancers identified. (**d**) Heatmap of correlation values between experimental replicate peak profiles resulting from unsupervised hierarchical clustering. A histogram in the upper left displays the frequency of correlation coefficients and the color of green shading associated with each coefficient (same shading associated with heatmap matrix). (**e**) Example (P23F-N1) peak plots showing refinement of broad peaks called in MACS2 (top) after HisTrader analysis for NFR regions (bottom). TSS = transcription start site, TES = transcription end site. (**f-o**) ΔΔCq values ($Cq_{experimental}$ – Cq_{GAPDH} from real-time ChIP-PCR to asses ten peaks identified from P30M-over-P30F analysis. (**p**) ΔΔCq values from a region of *MYH7B*, a gene with low levels of expression in brain. Bar plots show S.D. with symbols for each individual sample value.

a

P23 Male over P30 Male		
TFBS ID	MOTIF	P VALUE
MEF2A	TAAAAATA	9.63E-03

P30 Male over P23 Male		
TFBS ID	MOTIF	P VALUE
MEF2D	CTAAAATAG	2.58E-3
PRDM9	[illegible]	2.85E-3
ARX	TAATTA	3.67E-3
EN1	AATTA	3.98E-3
LHX4	TAATTA	4.13E-3
GBX2	AATTA	4.62E-3
HOXB4	TCATTA	6.63E-3
HOXD3	CTAATTA	6.72E-3
TFAP2B	GCC AGGC	7.86E-3
MEF2B	CTAAAATAG	8.98E-3
HOXC4	TCATTA	9.15E-3
NFIC	CTTGGCA	9.23E-3
ZNF263	GGGAGGA	9.77E-3

b

P23 Female over P30 Female		
TFBS ID	MOTIF	P VALUE
No enriched transcription factor binding sites		

P30 Female over P23 Female		
TFBS ID	MOTIF	P VALUE
ZNF148	CCCCTCCC	1.68E-3
FOSL1::JUND	TGAC	1.95E-3
Hoxd13	CAATAA	1.95E-3
PRDM9	[illegible]	2.37E-3
RFX7	GTTGCTA	3.06E-3
BATF	TGACTCA	3.35E-3
ZKSCAN1	AGTAGG	4.70E-3
ETV2::HOXB13	CGGA ATAA	4.70E-3
RORB	AGGTCA	5.02E-3
HOXB5	TAATTA	6.69E-3
BATF3	TGACTCA	7.83E-3
GRHL2	AACAGGTT	1.10E-2
FOSL2::JUN	ATGACGTCA	1.10E-2
BATF::JUN	ATGACTCA	1.17E-2

Supplementary Figure 4. Enrichment of TFBSs from differential H3K27ac peaks across age. (**a, b**) Enriched site (TFBS ID), motif, and p-value from comparison of P23M and P30M (**a**) and P23F and P30F (**b**) peak profiles.

		P23 Male		P23 Female		P30 Male		P30 Female	
TFBS ID	MOTIF	E VALUE	P VALUE	E VALUE	P VALUE	E VALUE	P VALUE	E VALUE	P VALUE
PRDM9	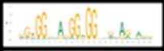	2.58E-19	3.07E-22	6.80E-8	8.08E-11	3.06E-12	3.65E-15	1.70E-3	2.03E-6
RFX5	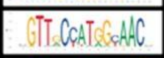	5.42E-19	6.44E-22	3.45E-5	4.10E-8	1.02E-13	1.21E-16	1.66E-3	1.98E-6
RFX3	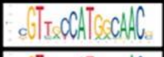	3.76E-15	4.48E-18	4.88E-7	5.80E-10	5.59E-9	6.67E-12	3.19E-3	3.81E-6
RFX2	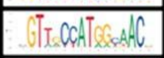	4.67E-12	5.56E-15	1.91E-6	2.27E-9	4.18E-11	4.98E-14	9.97E-5	1.19E-7
RFX1	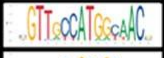	1.34E-13	1.60E-16	1.36E-6	1.61E-9	2.27E-9	2.70E-12	1.25E-3	1.49E-6
ZNF93	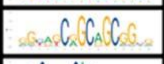	2.30E-10	2.74E-13	7.96E-4	9.46E-7	2.20E-7	2.63E-10	1.38E-2	1.65E-5
RREB1	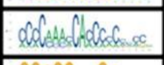	3.72E-8	4.43E-11	6.73E-4	8.00E-7	3.53E-3	4.22E-6	3.67	4.38E-3
ZNF281	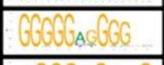	2.24E-4	2.66E-7	4.42E-3	5.26E-6	7.43E-3	8.86E-6	1.18E-4	1.40E-7
SP4	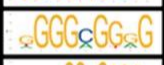	5.40E-6	6.42E-9	2.26E-1	2.69E-4	1.78E-3	2.13E-6	4.30E-3	5.13E-6
PATZ1	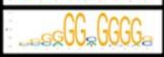	4.59E-6	5.46E-9	1.04E-1	1.24E-4	9.82E-5	1.17E-7	2.52	3.01E-3
RFX6	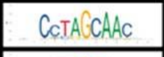	6.95E-8	8.27E-11	2.36	2.81E-3	1.25E-4	1.49E-7	7.11	8.48E-3
NFIC	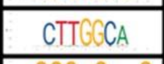	1.19E-6	1.41E-9	1.29	1.54E-3	2.11E-2	2.52E-5	3.65	4.36E-3
KLF1	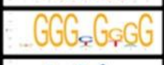	7.99E-7	9.50E-10	9.88	1.18E-2	3.49	4.16E-3	1.99E-2	2.38E-5
ZNF148	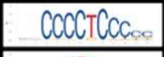	1.40E-4	1.67E-7	7.94	9.44E-3	9.55E-4	1.14E-6	5.10E-1	6.09E-4
SPIB	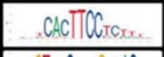	4.43E-6	5.27E-9	7.78	9.26E-3	1.42E-1	1.69E-4	4.00	4.77E-3
RFX4	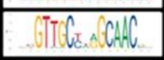	5.04E-3	5.99E-6	1.20	1.43E-3	3.05E-2	3.64E-5	4.47	5.33E-3
FOS	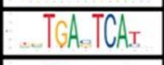	2.31E-2	2.74E-5	7.48E-1	8.89E-4	3.36E-1	4.01E-4	1.60	1.91E-3
CREB1	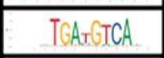	9.64E-4	1.15E-6	3.72	4.43E-3	1.43	1.71E-3	8.17	9.75E-3
FOSL2::JUND	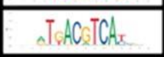	6.13E-3	7.29E-6	2.87	3.41E-3	6.26E-1	7.48E-4	7.66	9.14E-3
JUN::JUNB	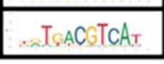	8.06E-2	9.58E-5	7.31	8.69E-3	2.74E	3.27E-3	6.98	8.33E-3

Supplementary Figure 5. Enrichment of TFBSs defined as the" core" set. Enriched site (TFBS ID), motif, and E- and p-value from the enrichment analysis for P23M, P23F, P30M, P30F.

a

TFBS ID	MOTIF	P23 Male E VALUE	P23 Male P VALUE	P30 Male E VALUE	P30 Male P VALUE
ATF2		6.63E-4	7.88E-7	2.50	2.98E-3
BATF		3.87E-1	4.61E-4	4.22	5.04E-3
BATF::JUN		3.22E-1	3.83E-4	3.96	4.72E-3
CREB3		2.11	2.51E-3	9.18E-1	1.10E-3
DBP		5.08E-2	6.04E-5	6.04	7.21E-3
EGR1		1.74E-2	2.07E-5	2.32E-1	2.76E-4
EGR2		8.09E-1	9.62E-4	8.28E-2	9.89E-5
FOSB::JUN		2.23E-3	2.65E-6	8.95E-2	1.07E-4
FOXC2		2.58	3.07E-3	1.19E-1	1.42E-4
HLF		6.14E-4	7.30E-7	4.97	5.93E-3
KLF11		1.30E-1	1.55E-4	5.45E-3	6.50E-6
KLF17		7.44E-5	8.85E-8	4.71E-3	5.62E-6
MEF2B		4.83E-1	5.74E-4	2.75E-1	3.28E-4
NR2C2		7.62E-1	9.06E-4	2.08	2.48E-3
NRF1		4.58	5.44E-3	1.82E-1	2.17E-4
POU2F1::SOX2		1.86	2.22E-3	3.83	4.57E-3
POU3F2		8.03	9.55E-3	9.95	1.19E-2
ZNF263		1.58	1.88E-3	4.20E-1	5.01E-4
ZNF701		4.54E-1	5.40E-4	1.58E-2	1.88E-5

b

TFBS ID	MOTIF	P23 Female E VALUE	P23 Female P VALUE	P30 Female E VALUE	P30 Female P VALUE
ATF4		2.99	3.56E-3	4.30E-1	5.13E-4
DMRTA2		1.12	1.33E-3	5.04	6.01E-3
DUX		8.95	1.06E-2	4.11E-1	4.90E-4
DUX4		2.67	3.17E-3	2.42E-2	2.88E-5
FOSL1::JUNB		1.52	1.81E-3	5.73	6.84E-3
NFIC::TLX1		1.63	1.94E-3	7.67E-1	9.15E-4
ONECUT1		4.89E-01	5.81E-4	5.29E	6.32E-3
ZNF382		4.84E-01	5.76E-4	1.22E	1.46E-3

Supplementary Figure 6. Enrichment of TFBSs from differential H3K27ac peaks after direct sex comparison. (**a, b**) Enriched site (TFBS ID), motif, and p-value from comparison of P23M and P23F (**a**) and P30M and P30F (**b**) peak profiles.
